# Genetic analysis of *Cryptozona siamensis* (Stylommatophora, Ariophantidae) populations in Thailand using the mitochondrial 16S rRNA and COI sequences

**DOI:** 10.1371/journal.pone.0239264

**Published:** 2020-09-14

**Authors:** Abdulhakam Dumidae, Pichamon Janthu, Chanakan Subkrasae, Wilawan Pumidonming, Paron Dekumyoy, Aunchalee Thanwisai, Apichat Vitta

**Affiliations:** 1 Department of Microbiology and Parasitology, Faculty of Medical Science, Naresuan University, Phitsanulok, Thailand; 2 Department of Helminthology, Faculty of Tropical Medicine, Mahidol University, Ratchathewi, Bangkok, Thailand; 3 Centre of Excellence in Medical Biotechnology (CEMB), Faculty of Medical Science, Naresuan University, Phitsanulok, Thailand; 4 Center of Excellence for Biodiversity, Faculty of Sciences, Naresuan University, Phitsanulok, Thailand; Universiti Malaysia Sabah, MALAYSIA

## Abstract

*Cryptozona siamensis*, one of the most widespread land snails, is native to Thailand, and plays a key role as an agricultural pest and intermediate host for *Angiostrongylus* spp. However, its genetic diversity and population structure has not yet been investigated, and are poorly understood. Therefore, a genetic analysis of the *C*. *siamensis* population in Thailand was conducted, based mitochondrial 16S rRNA (402 bp) and COI (602 bp) gene fragment sequences. *Cryptozona siamensis* randomly collected from 17 locations in four populations across Thailand, between May 2017 and July 2018. Fifty-eight snails were used to examine the phylogeny, genetic diversity, and genetic structure. The maximum likelihood tree based on the 16S rRNA and COI fragment sequences revealed two main clades. A total of 14 haplotypes with 44 nucleotide variable sites were found in the 16S rRNA sequences, while 14 haplotypes with 57 nucleotide variable sites were found in the COI sequences. The genetic diversity of *C*. *siamensis* in term of the number of haplotypes and haplotype diversity, was found to be high but the nucleotide diversity showed low levels of genetic differentiation for the COI sequence as also noted with the 16S rRNA sequence. The population genetic structure of *C*. *siamensis* revealed genetic difference in most populations in Thailand. However, low genetic difference in some populations may be due to high gene flow. This study provides novel insights into the basic molecular genetics of *C*. *siamensis*.

## Introduction

The land snail *Cryptozona siamensis*, a terrestrial pulmonate gastropod, belongs to the family Ariophantidae [1, 2]. *Cryptozona siamensis* have been reported as the intermediate host of *Angiostrongylus* that cause eosinophilic meningitis in human worldwide especially China, Taiwan, and Thailand [3, 4]. *Cryptozona siamensis* are important as intermediate hosts that promote the endemicity and transmission of *Angiostrongylus cantonensis* and *A*. *malaysiensis* [3, 5, 6]. The distribution of the snail hosts facilitates establishment of the life cycle of the parasite. In addition, the distribution of the land snails accelerates the spread of *A*. *cantonensis* [7, 8]. Ingestions of raw or undercooked infected snails, snails accidentally chopped up in vegetables, vegetable juices, salads, or foods contaminated by the slime of infected snails are highly risk factors for infection with *A*. *cantonensis* in human [9]. *Cryptozona siamensis* is native to Thailand, and is regarded as a cosmopolitan species, being one of the most widespread land snails in Southeast Asia [2, 10], with reports of *C*. *siamensis* from areas adjacent to Thailand, such as Malaysia, Singapore, and Laos [6, 11, 12]. *Cryptozona siamensis* has gained attention as an important agricultural and horticultural pest, in India, the United States of America, and Thailand [13, 14].

In the last few years, DNA sequencing data has been used to study and clarify the evolution of morphological characteristics of ambiguous organisms [15–18] while genetic studies of land snails using DNA sequence data could be valuable for their identification [19–21]. The most commonly used genes for genetic analysis in land snails are the mitochondrial cytochrome c oxidase subunit I (COI) and 16S rRNA genes [20, 22–24]. The COI genes have a greater range of phylogenetic information, compared with other mitochondrial genes, and are considered to be robust evolutionary markers for studies of inter-specific relationships [25, 26]. In addition, 16S rRNA gene has a high level of inter-specific polymorphisms. Hence, the 16S rRNA has been widely used in the genetic studies of snails [25, 27]. The genetic structure of the land snails has been studied from several geographical regions. In Hawaii, analysis of COI and 16S rRNA genes by haplotype networks, gene tree topologies, pairwise molecular divergence, and F_ST_ matrices revealed the substantial geographic genetic structuring and complex dispersal patterns of the land snail *Succinea caduca* [28]. In China, Zhou et al. studied population genetic structure of the land snail *Camaena cicatricosa* from 20 locations using mitochondrial gene (COI and 16S rRNA) and internal transcribed spacer (ITS2) sequences. This showed significant fixation indices of genetic differentiation and high gene flow among most populations [29]. In Thailand, the genetic variation of the COI in *Achatina fulica* was found to be low [30]. In Langkawi island of Malaysia, low genetic diversity of family Ariophantidae (*Cryptozona siamensis* and *Sarika resplendens*) and Dyakiiaea (*Quantula striata*) was also noted by using 16S rRNA gene [11].

Molecular population phylogeographic studies can offer information on specific genetic variations, population formations, and genetic structure [31]. Moreover, they can also help to identify how a population has been affected by various factors, including the ecological environment, climate, human activities, and geographical conditions [32–34]. However, the genetic diversity and structure of *C*. *siamensis* is currently poorly understood, as it has been the focus of only a limited number of studies. The genetic structure of *C*. *siamensis* from 3 populations of Thailand and additional one population from Malaysia was studied on allozyme variation on horizontal starch gel electrophoresis [2]. Although the genetic structure of *C*. *siamensis* was examined in previous study, no nucleotide sequencing of this land snail is available in the country. Therefore, the objective of this research was to investigate the genetic diversity and genetic structure of *C*. *siamensis* from Thailand, based on mitochondrial DNA-sequence variation at the COI and 16S rRNA loci.

## Methods

### Ethic and biosafety statement

The experimental protocol for the use of animals (snail intermediate host) in this study was approved by the Center for Animal Research of Naresuan University (Project Ethics Approval No: NU-AQ610711). The biosafety protocol was approved by the Naresuan University Institutional Biosafety Committee (Project Approval No: NUIBC MI 61-08-50).

### Collection and preliminary identification of the snails

**Fig 1:**
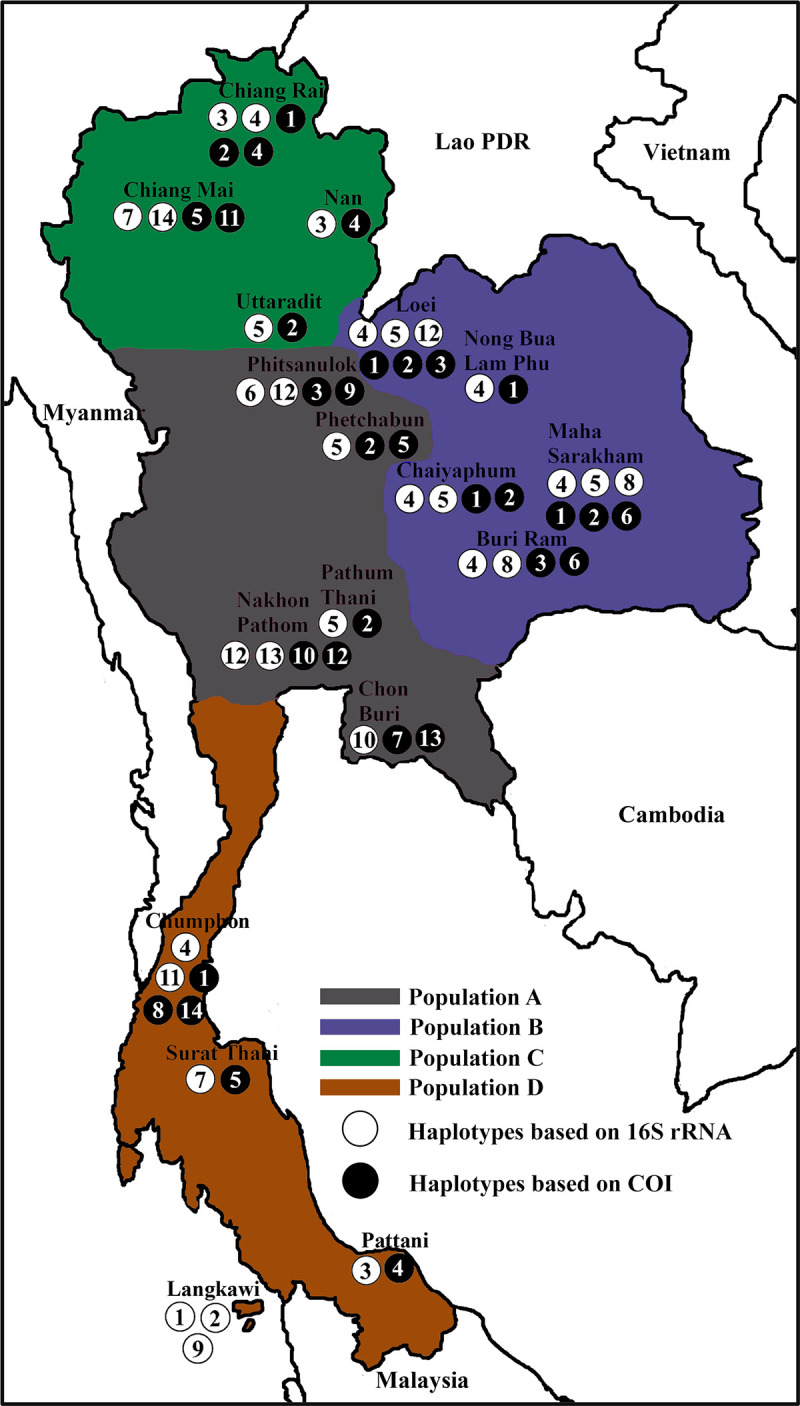
Geographical locations of the 4 populations of *C*. *siamensis* in Thailand, with the 14 haplotypes based on the 16S rRNA and COI sequences. Numbers inside the closed and opened circles indicates the haplotype number.

During a survey for *A*. *cantonensis*, which uses snails as an intermediate host, *C*. *siamensis* specimens were randomly collected from 17 locations across Thailand, between May 2017 and July 2018 (Fig 1). The snail populations (A, B, C, and D) were defined according to the biogeographical regions (Table 1). The snails were located in several types of natural habitats, such as under or above trunks of fallen trees, under stones, in the pot of flower, and in wall crevices. The snails were collected by hand picking and then put in a net with air for transporting to the Department of Microbiology and Parasitology, Faculty of Medical Science, Naresuan University, Phitsanulok, Thailand. All snails were cleaned with tap water and were preliminary identified according to the previously recorded morphological description of *C*. *siamensis* [12]. These included having medium sized shell (> 30 mm in shell width) that was two-toned in color, a surface with fine reticulate sculptures a discoidal shell with a low spire, light straw colored ventral part, light brown dorsal part, a fine surface with fine dense axial grooves, and a smooth ventral surface, and is slightly shiny [12]. The body of each *C*. *siamensis* specimen was removed from its shell, and approximately 25 mg of foot tissue was removed and preserved at -20°C for subsequent DNA extraction. To detect the *Angiostrongylus* larvae, the remaining snail tissue was artificially digested with a 0.7% (w/v) pepsin solution, as previously described [3]. Fortunately, all the *C*. *siamensis* samples in this study were found to be negative for *Angiostrongylus* larvae.

**Table 1:** Sampling locations of the *Cryptozona siamensis* collected from across Thailand, with the 14 haplotypes based on the 16S rRNA and COI sequences.

Population	Province	Latitude	Longitude	Sample Name	16S rRNA haplotype	COI haplotype
A	Phitsanulok	16.821123	100.265851	Phitsanulok 1	16S12	CO3
				Phitsanulok 2	16S12	CO3
				Phitsanulok 3	16S12	CO3
				Phitsanulok 4	16S6	CO9
	Phetchabun	16.301669	101.119280	Phetchabun 1	16S5	CO2
				Phetchabun 2	16S5	CO2
				Phetchabun 3	16S5	CO5
				Phetchabun 4	16S5	CO2
	Pathum Thani	14.020839	100.525027	Pathum Thani 1	16S5	CO2
				Pathum Thani 2	16S5	CO2
				Pathum Thani 3	16S5	CO2
				Pathum Thani 4	16S5	CO2
	Nakhon Pathom	13.819920	100.062167	Nakhon Pathom 1	16S13	CO10
				Nakhon Pathom 2	16S12	CO12
				Nakhon Pathom 3	16S12	CO12
				Nakhon Pathom 4	16S12	CO12
	Chon Buri	13.361143	100.984671	Chon Buri 1	16S10	CO7
				Chon Buri 2	16S10	CO7
				Chon Buri 3	16S10	CO13
B	Loei	17.486023	101.722300	Loei 1	16S4	CO1
				Loei 2	16S4	CO1
				Loei 3	16S12	CO3
				Loei 4	16S5	CO2
	Nong Bua Lam Phu	17.221824	102.426036	Nong Bua Lam Phu 1	16S4	CO1
				Nong Bua Lam Phu 2	16S4	CO1
				Nong Bua Lam Phu 3	16S4	CO1
				Nong Bua Lam Phu 4	16S4	CO1
	Chaiyaphum	15.806817	102.031502	Chaiyaphum 1	16S4	CO1
				Chaiyaphum 2	16S5	CO2
				Chaiyaphum 3	16S4	CO1
				Chaiyaphum 4	16S5	CO2
	Maha Sarakham	16.013201	103.161516	Maha Sarakham 1	16S8	CO6
				Maha Sarakham 2	16S8	CO6
				Maha Sarakham 3	16S4	CO1
				Maha Sarakham 4	16S5	CO2
				Maha Sarakham 5	16S5	CO2
	Buri Ram	14.993001	103.102919	Buri Ram 1	16S4	CO1
				Buri Ram 2	16S4	CO1
				Buri Ram 3	16S8	CO6
				Buri Ram 4	16S4	CO1
C	Chiang Rai	19.907165	99.830955	Chiang Rai 1	16S3	CO4
				Chiang Rai 2	16S3	CO2
				Chiang Rai 3	16S4	CO1
	Chiang Mai	18.706064	98.981716	Chiang Mai 1	16S14	CO11
				Chiang Mai 2	16S7	CO5
	Nan	18.775631	100.773041	Nan 1	16S3	CO4
				Nan 2	16S3	CO4
				Nan 3	16S3	CO4
	Uttaradit	17.620088	100.099294	Uttaradit 1	16S5	CO2
D	Chumphon	10.493049	99.180019	Chumphon 1	16S11	CO8
				Chumphon 2	16S11	CO14
				Chumphon 3	16S4	CO1
				Chumphon 4	16S11	CO8
				Chumphon 5	16S4	CO1
	Surat Thani	9.138238	99.321748	Surat Thani 1	16S7	CO5
				Surat Thani 2	16S7	CO5
				Surat Thani 3	16S7	CO5
	Pattani	6.761830	101.323254	Pattani 1	16S3	CO4
Total	17			58		

### Genomic DNA extraction

Genomic DNA from each individual *C*. *siamensis* was extracted using a Tissue & Cell Genomic DNA Purification Kit (GMbiolab Co., Ltd. Taichung, Taiwan), according to the manufacturer’s instructions. An aliquot of the DNA solution was checked by running it on a 0.8% (w/v) agarose gel, in a 1 × TBE buffer, at 100 V. The gel was stained with ethidium bromide, followed by a destaining with distilled water, and photographed under UV light. The rest of the DNA solution was kept at -20°C for later use as the PCR template.

### Polymerase chain reaction (PCR) and sequencing

The DNA fragment (500 bp) of the 16S rRNA gene was amplified by PCR using the 16Sar (5′-CGCCTGTTTATCAAAAACAT-3′) and 16Sbr (5′-CCGGTCTGAACTCAGATCACGT-3′) primer [35]. The PCR amplifications were performed in a 30 μl total volume, containing 15 μl of EconoTaq^®^ PLUS 2 × Master mix (1×; Lucigen Corporation, Middleton, WI, USA), 1.5 μl of 5 μM of each primer (0.25 μM), 9 μl of distilled water, and 3 μl of the DNA template (20–200 ng). Thermal cycling for the 16S rRNA PCR amplification was performed at 96°C for 2 min, followed by 35 cycles of 94°C for 30 s, 45°C for 1 min, and 72°C for 2 min, and then a final 72°C for 5 min [36]. The 710 bp segment of the COI gene was amplified using the LCO1490 (5′-GGTCAACAAATCATAAAGATATTGG-3′) and HCO2198 (5′-TAAACTTCAG GGTGACCAAAAAATCA-3′) primer [37] as described for the 16S rRNA except the thermal cycling used annealing and extension time of 40 s and 90 s, respectively. All PCR amplifications were conducted in a Biometra TOne Thermal cycler (Analytik Jena AG, Jena, Germany). The amplified products were analyzed by 1.2% (w/v) agarose gel-electrophoresis at 100 V, stained with ethidium bromide, destained with distilled water, and visualized and photographed under UV light. The PCR products were then purified using a NucleoSpin^®^ Gel and a PCR Clean-up kit (Macherey-Nagel, Germany), according to the manufacturer’s instructions. An aliquot of the purified PCR product was checked by 1.2% (w/v) agarose gel electrophoresis as above, while the rest was used as the template for commercial sequencing (Macrogen Inc., Seoul, Korea) in both the forward and reverse directions using the same primers as in the PCR.

### Sequence and phylogenetic analysis

The nucleotide sequences were edited by viewing the peaks of the chromatogram in the SeqMan II software (DNASTAR, Madison, WI, USA). Phylogenetic analysis, including species identification (conversion of molecular operational taxonomic unit to likely species designation) of the *Cryptozona* was performed by BLASTn searching the NCBI database (http://blast.ncbi.nlm.nih.gov/Blast.cgi) an aligning the obtained homologous nucleotide sequences using ClustalW. Phylogenies were estimated using maximum likelihood (ML) with the general time reversible model, neighbor joining (NJ) with Kimura two-parameter (K2), and maximum parsimony (MP) with the subtree-pruning-regrafting (SPR) and node support values, based on 1000 bootstrap replicates, in the MEGA Version 7.0 program [38]. In addition, Bayesian inference (BI) analysis was performed using MrBayes version 3.2 [39], where the tree space was explored, using four chains for each run of a Markov chain Monte Carlo algorithm (MCMC). The BI analysis was run for 10 000 000 generations and sampled every 100 generations. The last 10 000 trees were used for the Bayesian posterior probabilities (bpp) with a burnin of 90 001 samples, as previous reported [40]. Although, four methods were used for constructing a phylogeny, only ML topology was showed in the present study. The bootstrap values from three methods and the percentage of Bayesian posterior probabilities were indicated on the branch of ML tree.

### Genetic analysis

Haplotype diversity and nucleotide diversity were calculated in ARLEQUIN, version 3.5.1.2 [41]. The relationships among the haplotypes were estimated using the median joining (MJ) network [42]. The MJ network analysis was performed in NETWORK, version 5.0.1.1, based on 65 sequences of 16S rRNA (includes 7 sequences from GenBank) and 58 sequences of COI sequences.

The genetic differentiation among the populations from each region, was calculated in ARLEQUIN, based on pairwise *F*_ST_. Analysis of molecular variance (AMOVA) performed in ARLEQUIN was used to test the genetic difference among groups. Although the data were generated for 16S rRNA and COI gene fragments, for most of the populations from the different regions, the substitution rate was higher for the COI, making it the more appropriate and informative population level marker. Thus, the COI data were the focus of the population genetic structure analyses.

## Results

### Molecular identification of *Cryptozona siamensis*

To identify the *Cryptozona* species, 58 individual land snails (19 samples from population A, 21 samples from population B, 9 samples from population C, and 9 samples from population D) were randomly selected for genetic studies. PCR-based analysis and sequencing of their 16S rRNA and COI regions, was performed, together with a BLASTN search of the edited sequences. Based on the 402 bp of the 16S rRNA gene, all 58 sequences (GenBank accession nos. MK858467-MK858524) in this study showed high identity (96 to 99%) with those of GenBank accession no. JQ728565, which is in agreement with the identification of the taxa in the present study as *C*. *siamensis*.

### Phylogeny of *Cryptozona siamensis*

**Fig 2:**
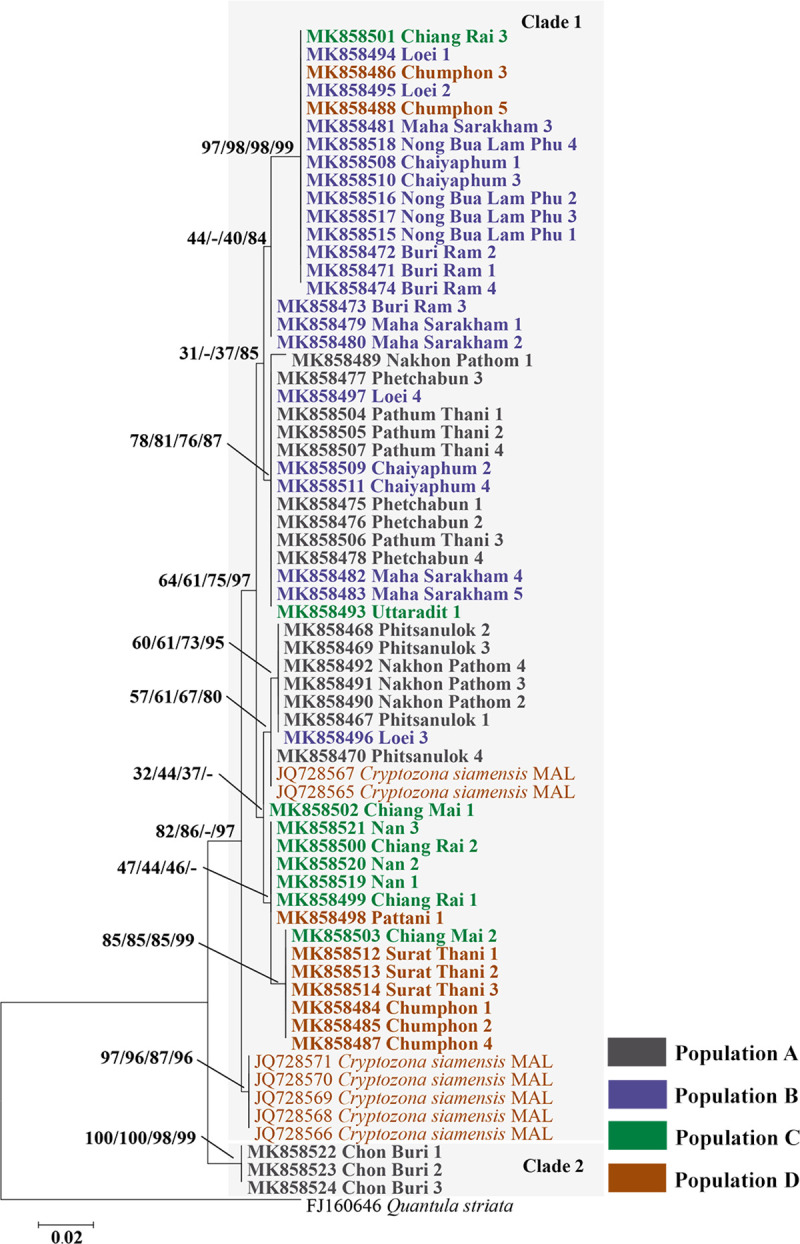
Maximum likelihood phylogenetic tree generated from 58 sequences of a partial 16S rRNA sequence (402 bp) of *C*. *siamensis*, collected from across Thailand. Support values (ML bootstrap/NJ bootstrap/MP bootstrap/Bayesian posterior probabilities) are shown above the branches. Bold letters indicate the sequences obtained in the present study. *Quantula striata* was used as the out-group. MAL = Malaysia.

A phylogenetic tree was constructed using the ML, NJ, MP, and BI methods, and the 58 sequences, based on their 16S rRNA genes, were divided into two main clades. Based on the topology of ML tree (Fig 2), clade 1 (from all populations) contained 55 of the sequences, and they were sourced from Phitsanulok, Phetchabun, Pathum Thani, Nakhon Pathom, Loei, Nong Bua Lam Phu, Chaiyaphum, Maha Sarakham, Buri Ram, Chiang Rai, Chiang Mai, Nan, Uttaradit, Chumphon, Surat Thani, and Pattani Provinces. These sequences were closely related to 7 sequences of *C*. *siamensis* from Malaysia with 97% of Bayesian posterior probability. Clade 2 contained 3 sequences from the Chon Buri Province (Fig 2), with 100% bootstrap support values for each ML and NJ method. The intraspecific distances among the samples were 0.0–5.4% (S1 Table).

**Fig 3:**
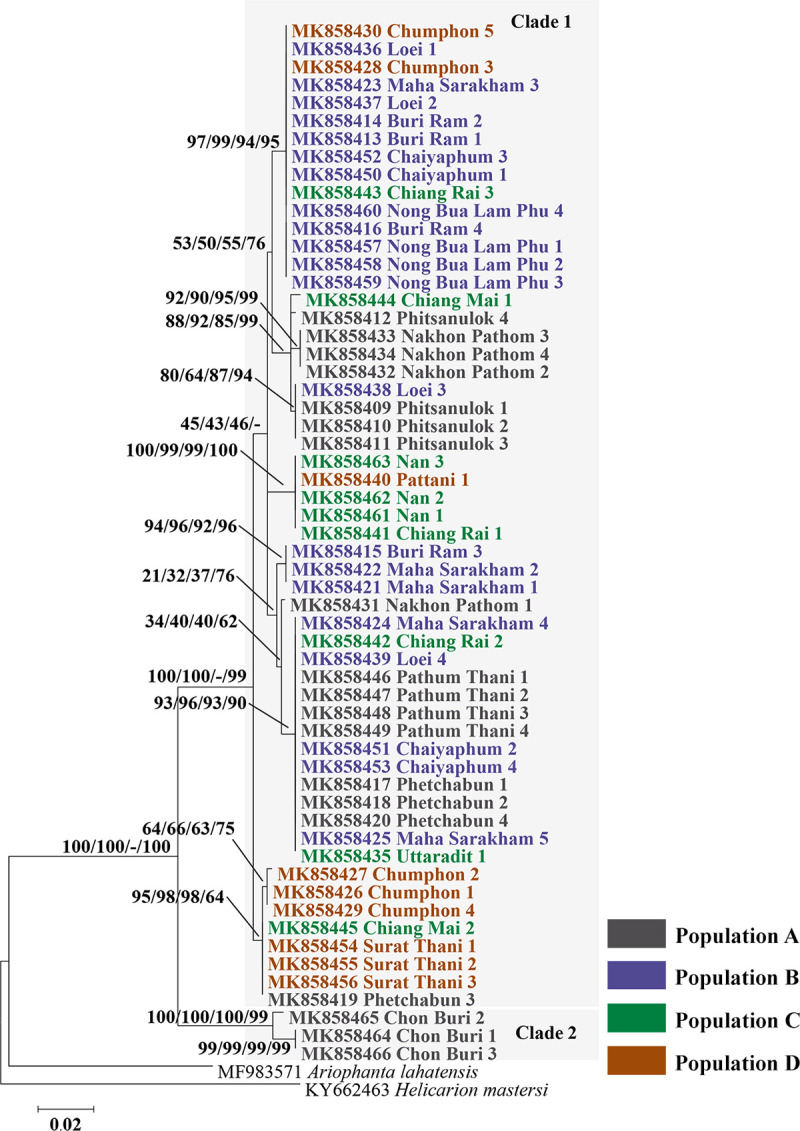
Maximum likelihood phylogenetic tree generated from 58 sequences of a partial COI sequence (602 bp) of *C*. *siamensis*, collected from across Thailand. Support values (ML bootstrap/NJ bootstrap/MP bootstrap/Bayesian posterior probabilities) are shown above the branches. Bold letters indicate the sequences obtained in the present study. *Ariophanta lahatensis* and *Helicarion mastersi* were used as the out-group.

The phylogenetic analysis based on the COI sequences (602 bp), for the 58 *C*. *siamensis* samples (GenBank accession nos. MK858409-MK858466), revealed 2 main clades (Fig 3). Clade 1 contained 55 sequences with highest bootstrap support values for ML and NJ of 100% and 100%, respectively. Clade 2 contained 3 sequences where the bootstrap support values for ML, NJ, and MP were 100% for each method. Intraspecific distances among the samples were 0.0–7.5% (S2 Table).

### Mitochondrial DNA sequence variation

**Fig 4:**
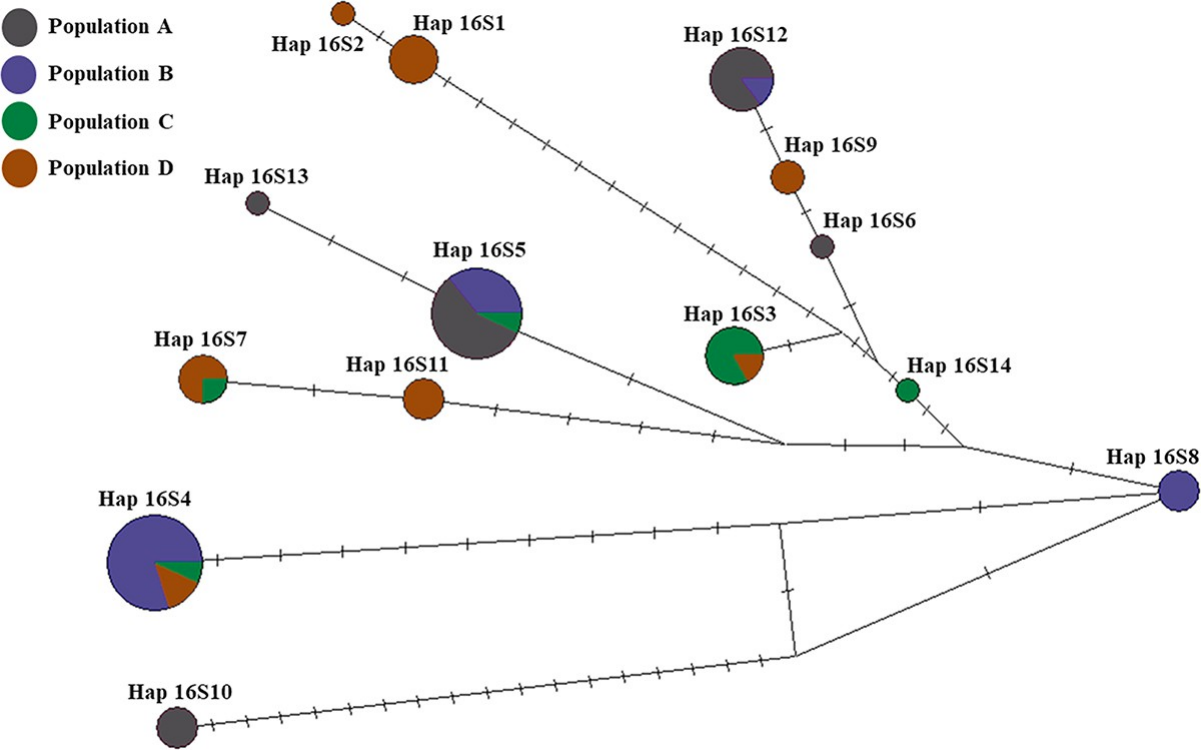
Mitochondrial DNA genealogy for 65 16S rRNA sequences (58 sequences from Thailand and 7 sequences from Malaysia) from *C*. *siamensis*, constructed using a median joining network method. Each haplotype is represented by a circle. Sizes of the circles are relative to the number of individuals sharing specific haplotypes. Each mutation between the haplotypes is represented by a bar.

The mitochondrial 16S rRNA gene (402 bp) was obtained from 58 sequences of *C*. *siamensis* in Thailand and 7 sequences from Malaysia in 4 populations (A, B, C, and D). Fourteen haplotypes (16S1-16S14) were identified with 44 nucleotide variation sites (S3 Table). Of these, 9 haplotypes (16S1, 16S2, 16S6, 16S8, 16S9, 16S10, 16S11, 16S13, and 16S14) were unique, and 5 haplotypes were shared by at least two populations (Table 1 and S4 Table). The geographically wide spread haplotype 16S3, is shared between the populations C and D. Haplotype 16S4 was shared between the populations B, C, and D. Haplotype 16S5 was shared between the populations A, B, and C. Haplotype 16S7 was shared between the populations C and D. Haplotype 16S12 was shared between the populations A and B (Figs 1 and 4). The haplotype diversity in each population ranged from 0.6238 in population B to 0.8833 in population D, with a mean of 0.8779. Nucleotide diversity in each population ranged from 0.0088 in population C to 0.0169 in population A, with a mean of 0.0169 (Table 2).

**Table 2:** Haplotype and nucleotide diversity of 4 populations of *Cryptozona siamensis* identified in Thailand and Malaysia, based on their mitochondrial 16S rRNA sequences.

Populations	Number of samples	No. haplotype	Haplotype diversity (*h*), mean ± SD	Nucleotide diversity (*π*), mean ± SD
A	19	5	0.7310±0.0663	0.0169±0.0093
B	21	4	0.6238±0.0899	0.0119±0.0067
C	9	5	0.7222±0.1592	0.0088±0.0056
D	16	7	0.8833±0.0452	0.0155±0.0087
Total	65	14	0.8779±0.0215	0.0169±0.0089

**Fig 5:**
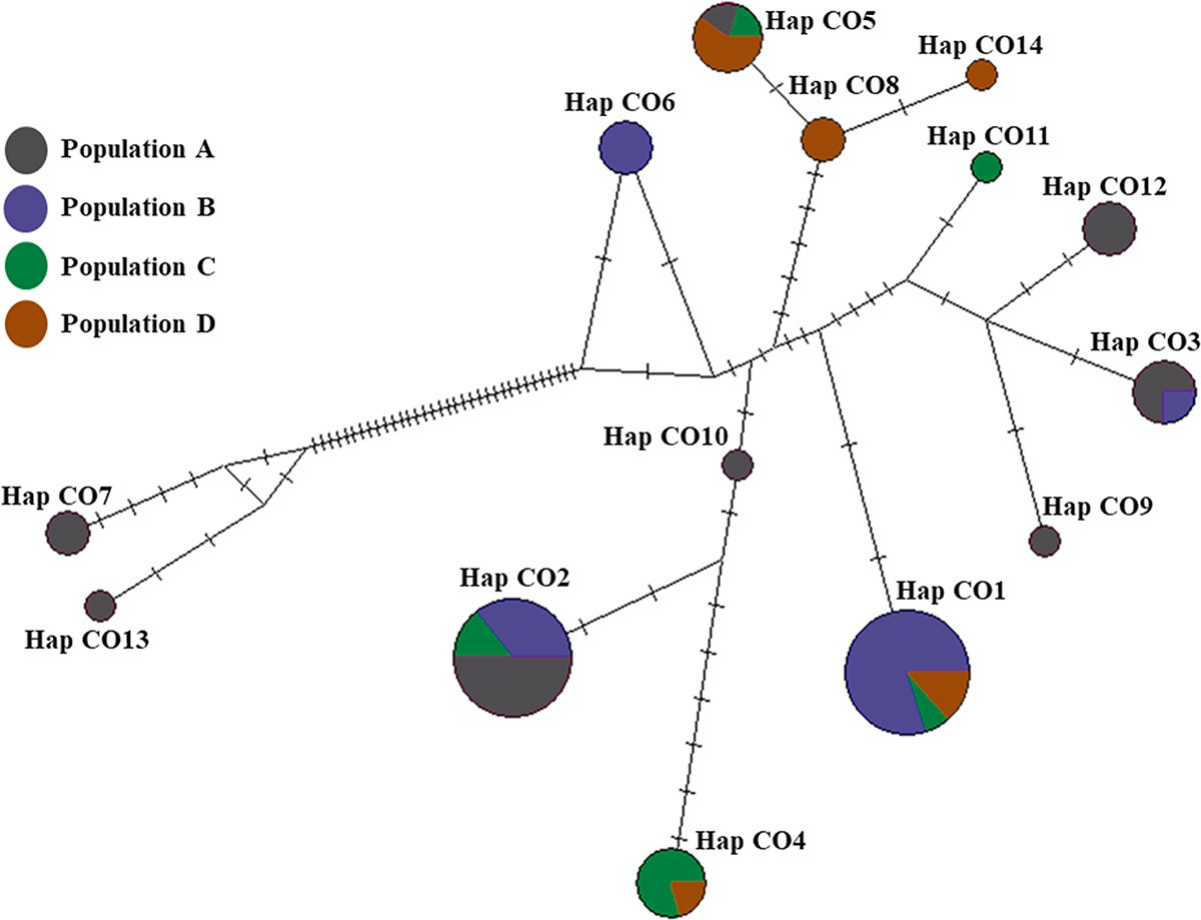
Mitochondrial DNA genealogy for 58 COI sequences of *C*. *siamensis* constructed using a median joining network method. Each haplotype is represented by a circle. Sizes of the circles are relative to the number of individuals sharing specific haplotypes. Each mutation between the haplotypes is represented by a bar.

Fourteen haplotypes (CO1-CO14), from the 58 sequences of the 4 populations (A, B, C, and D) in Thailand, were identified, based on the COI genes (602 bp) with nucleotide variations at 57 sites (S5 Table). Of these, 9 haplotypes (CO6, CO7, CO8, CO9, CO10, CO11, CO12, CO13, and CO14) were unique, and 5 haplotypes were shared by at least two populations (Table 1 and S6 Table). The geographically spread haplotype CO1 was shared between the population B, C, and D. Haplotype CO2 was shared between the populations A, B, and C. Haplotype CO3 was shared between the populations A and B. Haplotype CO4 was shared between the populations C and D. Haplotype CO5 was shared between the populations A, C, and D (Figs 1 and 5). The haplotype diversity in each population ranged from 0.6238 in population B to 0.8611 in population D, with a mean of 0.8609. Nucleotide diversity in each population ranged from 0.0083 in population B to 0.0271 in population A, with a mean of 0.0180 **(Table 3)**.

**Table 3:** Haplotype and nucleotide diversity of 4 populations of *Cryptozona siamensis* in Thailand, based on their mitochondrial cytochrome c oxidase subunit I gene sequences.

Populations	Number of samples	No. haplotype	Haplotype diversity (*h*), mean ± SD	Nucleotide diversity (*π*), mean ± SD
A	19	8	0.8363±0.0651	0.0271±0.0141
B	21	4	0.6238±0.0899	0.0083±0.0047
C	9	5	0.8056±0.1196	0.0131±0.0076
D	9	5	0.8611±0.0872	0.0098±0.0059
Total	58	14	0.8609±0.0271	0.0180±0.0092

### Population genetic structure

Population pairwise *F*_ST_ values for the 16S rRNA sequences of the *C*. *siamensis*, revealed statistically significant differentiation (P < 0.05), whereas between the populations C and D no genetic differences were identified (Table 4). The population pairwise *F*_ST_ values based on the COI sequences, between the populations A, B, C, and D were significantly different genetically (Table 5).

**Table 4:** Population pairwise *F*_ST_, between the 4 populations of *Cryptozona siamensis* in Thailand and Malaysia, based on the mitochondrial 16S rRNA gene sequences.

Populations	A	B	C	D
A	-			
B	0.334*	-		
C	0.177*	0.345*	-	
D	0.175*	0.301*	0.059	-

* P<0.05.

**Table 5:** Population pairwise *F*_ST_ between 4 populations of *Cryptozona siamensis* in Thailand, based on the mitochondrial cytochrome c oxidase subunit I gene sequences.

Populations	A	B	C	D
A	-			
B	0.170*	-		
C	0.094*	0.204*	-	
D	0.180*	0.313*	0.215*	-

* P<0.05.

## Discussion

We have analyzed the genetics of the land snail *C*. *siamensis*, based on its mitochondrial 16S rRNA and COI genes. *Cryptozona siamensis* showed high haplotype diversity (16S rRNA mean haplotype diversity = 0.8779 and the COI gene mean haplotype diversity = 0.8609). The 65 16S rRNA gene sequences, were classified into 14 haplotypes, 9 that were unique, and 5 that were shared between the different populations. The 58 COI sequences were classified into 14 haplotypes, 9 that were unique and 5 that were shared between the different populations. High haplotype diversity (39 mitochondrial haplotypes) was also reported from *Camaena cicatricose* in China [29]. In contrast, low haplotype diversity (2 COI haplotypes) was found in *A*. *fulica* in Thailand [30]. The genetic diversity of the 16S rRNA and the COI genes of the *C*. *siamensis*, were different. These results were similar to those from a previous report on the genetic diversity of the 16S rRNA and COI genes of the land snail *Cyclophorus fulguratus* [40]; as the genetic variation in the 16S rRNA gene was lower than that of the COI gene. This suggested that the 16S rRNA gene in the land snail was a slowly evolving region of the mtDNA [43]. In terms of the evolution rate, the COI gene was superior to the 16S rRNA gene [44], and thus considered to be a reliable phylogenetic marker for the *C*. *siamensis*. Many previous reports in land snails have established that the COI gene is a reliable molecular marker for phylogenetic analysis [44–46]. In contrast with the haplotype diversity, the nucleotide diversity was low for both the 16S rRNA (mean = 0.0169) and COI (mean = 0.0180) genes for this snail. The low nucleotide diversity of *C*. *siamensis* in the present study could be due to small sample sizes. This was consistent with the previous research on the *Succinea cadusa* snail [28]. The pattern of high haplotype diversity and low nucleotide diversity implies that independent founder events have resulted in multiple unique populations, that each persisted in isolation for sufficient amounts of time, as to allow the accumulation of substitutions through drift [28]. The genetic diversity of the species could be affected by mutation and selection. These effects may play an important role in populations with genetic diversity. In addition, the climate may effect on the population size. This could be observed in other snails such as *Camaena cicatricosa* and *Succinea caduca* [28, 29]. In addition, the genetic diversity of the *Collisella subrugosa* snail in Brazil showed variation due to environmental conditions and differing selection pressures [47].

The population genetic structure was examined using the COI and 16S sequence data from 4 populations of Thailand. The substitution rate for the COI gene was high in the *C*. *siamensis* from all populations. Previous research has suggested that when the pairwise *F*_ST_ is greater than 0.15, it implies a high level of genetic differentiation among the population. Whereas, if the pairwise *F*_ST_ is between 0 and 0.05, it implies a low level of genetic differentiation between the populations [48]. In this study, the population genetic structure of the pairwise *F*_ST_ values showed a significant genetic differentiation between the most populations, based on the analysis of the 16S rRNA and COI genes. Moreover, the phylogenetic analysis and haplotype network construction showed the lack of clear population genetic structures. This suggests that the gene flow within the *C*. *siamensis* population in Thailand might bring about genetic homogeneity [49]. In this study, almost all of the pairwise *F*_ST_ values, based on the 16S rRNA and COI sequences between the populations, were over 0.15. In contrast, the pairwise *F*_ST_ based values on the 16S rRNA sequences, showed different results in some populations compared to the COI sequences. This suggested that the 16S rRNA gene had a relatively slow of evolution rate, and a low variability, compared to the COI gene [44, 50]. This agreed with the study of Feng et al. (2011), who found more variable nucleotide sites in the COI gene than in the 16S rRNA in the mollusk of the family Pectinidae. Genetic differentiation of *C*. *siamensis* population may be affected by the ecological environment, climate barriers, and geographic barrier factors [2, 29]. This was supported by previous studies that showed that the ecological environment factor on population differentiation, has been reported in other land snails, such as *Camaena cicatricose* [29] and *Cyclophorus fulguratus* [42, 51]. In addition, the migration and expansion repeated of population from different areas lead to continuously mix the populations of land snails. It is likely to affect accumulation of high genetic diversity within the population of the species. This was reported in pulmonate snail, *Euhadra quaesita* [52]. It may be possible reason leading to the genetic different in population of *C*. *siamensis*.

The genetic homogeneity of *C*. *siamensis* in Thailand based on 16S rRNA and COI genes was found among some populations. The results suggested that extensive gene-flow was a possibility. In support of this, considerable levels of gene-flow have been reported among *C*. *siamensis* populations in Thailand. Populations of *C*. *siamensis* in the areas with a low level of genetic differentiation, exhibit higher levels of gene flow than the populations with a high genetic differentiation [53]. Although this particular land snail has a low dispersal ability, the water, wind, anthropochory, and other factors, can lead to a wider distribution, especially for human activities [54]. Recently, Prasankok and Panha (2011) reported on the allozyme variations in the *C*. *siamensis* from the three regions of Thailand, and one region in Malaysia. The population of *C*. *siamensis* among the 3 geographic regions (north, central, and south) of Thailand as well as the population in Malaysia, showed a high degree of gene flow. This was consistent with the research that the haplotype network of *C*. *siamensis* in Thailand was shared and linked to the haplotype from Malaysia with mutation steps. In the present study, gene flow within the *C*. *siamensis* population in Thailand could be possible for several reasons. *Cryptozona siamensis* often occurred in habitats associated with human activities, such as vegetable gardens, in flower pots, and in wall crevices of houses. In particular, the transportation of potted plants or vegetables contaminated with snails across provinces may have promoted the movement of snails. Therefore, the possibility of dispersal of *C*. *siamensis* among populations in each area by humans could be plausible. Similarly, the African land snail in China, *A*. *fulica*, could be spread through shipments of plants especially pot plants [55], as well as the *C*. *cicatricosa* in China could be spread through cargo transportation [29]. Therefore, the dispersal of *C*. *siamensis* between populations in each region may arise by human activities [2]. The possibility of gene flow may be associated with the anthropochoric effects of snail dispersal [2, 56]. In addition, birds perhaps, mediated important in the transport of snails [57]. The movements of the eggs or small snails attached to the birds may have been important in the distributions between each area in regions. Thus, it is suggested that gene flow in the *C*. *siamensis* from Thailand may be explained using several possibilities. In addition, transportation by human activities may promote the spread of *A*. *cantonensis* which is hosted by this land snail. Similarly, Lv et al. (2009) reported the distribution of *A*. *cantonensis* in China associated with invasive land snail, *A*. *fulica* [8]. Although no *C*. *siamensis* infected with *A*. *cantonensis* was found in the present study, previous reports found that *C*. *siamensis* was infected with *A*. *cantonensis*. In Thailand, *C*. *siamensis* infected with *A*. *cantonensis* was found in Phetchabun, Kalasin, Phitsanulok, and Kamphaeng Phet provinces [3]. In general, food made from *C*. *siamensis* are uncommon dish for people in Thailand. Human may get infection with *A*. *cantonensis* by consumption of vegetable contaminated with *A*. *cantonensis* larvae [58]. Therefore, the *C*. *siamensis* is important to maintain the life cycle of *A*. *cantonensis* and may transmit to human.

## Conclusion

We have reported on the genetic diversity of the 16S rRNA and the COI genes, from the *C*. *siamensis* samples taken in Thailand. The maximum likelihood tree based on the 16S rRNA and COI fragment sequences of *C*. *siamensis* revealed two main clades. Most of the sequences fell in clade 1 and 3 samples from Chon Buri Province (population A) was placed in clade 2. The genetic diversity of *C*. *siamensis* in term of the number of haplotypes and haplotype diversity, was found to be high but the nucleotide diversity among the different populations of Thailand showed low levels of genetic differentiation for the COI sequence as also noted with the 16S rRNA sequences. Population genetic structure of *C*. *siamensis* based on *F*_ST_ value in 16S rRNA and COI genes was genetically different among most of population exception of some populations. Genetic differentiation in the populations of the *C*. *siamensis* may result from the effects of the ecological environment, climate barriers, and geographic barriers. However, low genetic difference in some populations may be due to high gene flow which may occur from the transportation by human activities. In addition, transportation by human may lead to spread of *A*. *cantonensis* which is hosted by *C*. *siamensis*. This study shows the genetic diversity of the *C*. *siamensis* populations across several regions of Thailand, and their interrelatedness.

## Supporting information

S1 Table Pairwise p-distances 16S rRNA gene within *C*. *siamensis* samples. (XLS)

S2 Table Pairwise p-distances COI gene within *C*. *siamensis* samples. (XLS)

S3 Table Forty-four variable sites across the 14 haplotypes of *C*. *siamensis* based on the 16S rRNA sequences. (PDF)

S4 Table Haplotype frequency based on 16S rRNA sequences in each population. (PDF)

S5 Table Fifty-seven variable sites across the 14 haplotypes of *C*. *siamensis* based on COI sequences. (PDF)

S6 Table Haplotype frequency based on COI sequences in each population. (PDF)
