# Supplementary material for: Genetic analysis of Cryptozona siamensis (Stylommatophora, Ariophantidae) populations in Thailand using the mitochondrial 16S rRNA and COI sequences
Source: PLoS One. 2020 Sep 14;15(9):e0239264. doi: 10.1371/journal.pone.0239264 (PMC7489551; doi:10.1371/journal.pone.0239264)
Supplement: S3 Table — (PDF) [file pone.0239264.s003.pdf]

S3 Table. Forty-four variable sites across the 14 haplotypes of *C. siamensis* based on the 16S rRNA sequences.

Haplotype	Nucleotide positions																																										
	**28**	**54**	**73**	**74**	**75**	**80**	**85**	**100**	**102**	**107**	**135**	**142**	**172**	**202**	**203**	**204**	**205**	**206**	**207**	**208**	**209**	**210**	**211**	**212**	**215**	**216**	**217**	**218**	**219**	**220**	**221**	**222**	**223**	**226**	**229**	**247**	**249**	**251**	**253**	**265**	**312**	**313**	**321**	**353**
16S1	T	A	C	C	A	T	G	C	A	T	T	A	T	A	C	A	T	C	A	A	C	A	T	A	A	C	T	T	T	G	T	G	T	A	-	G	T	A	A	A	-	-	G	G
16S2	-	A	C	C	A	T	G	C	A	T	T	A	T	A	C	A	T	C	A	A	C	A	T	A	A	C	T	T	T	G	T	G	T	A	-	G	T	A	A	A	-	-	G	G
16S3	T	A	C	A	A	T	G	C	A	A	T	A	T	G	C	A	T	-	-	-	-	-	-	A	A	T	T	T	T	T	T	G	T	A	-	G	T	A	A	G	-	-	G	A
16S4	T	A	C	C	A	T	A	T	A	A	A	G	T	G	C	A	A	-	-	-	-	-	-	T	-	-	-	-	T	T	G	G	T	A	-	G	T	G	A	G	T	T	G	A
16S5	T	A	C	C	A	T	G	C	A	A	A	A	T	A	C	A	T	-	-	-	-	-	-	A	-	-	-	-	-	T	T	G	T	A	-	G	T	A	A	G	-	-	A	A
16S6	T	A	C	C	A	T	G	C	A	A	T	A	T	G	C	A	T	-	-	-	-	-	-	A	A	T	-	-	T	T	T	G	T	A	-	A	T	A	A	G	-	-	G	A
16S7	T	A	C	A	A	C	G	C	A	A	A	A	T	G	C	A	T	-	-	-	-	-	-	A	-	-	-	-	-	T	T	G	C	A	A	G	T	A	A	G	-	-	G	A
16S8	T	A	C	C	A	T	A	C	A	A	A	A	T	A	C	A	T	-	-	-	-	-	-	A	A	-	-	-	T	T	T	G	T	A	-	G	T	A	A	G	-	-	G	A
16S9	T	A	C	C	A	T	G	C	A	A	T	A	T	G	C	A	T	-	-	-	-	-	-	A	A	T	-	-	T	T	-	G	T	A	-	A	T	A	A	G	-	-	G	A
16S10	T	G	T	C	C	T	A	C	T	T	T	A	-	A	G	G	C	A	-	-	-	-	-	A	A	-	-	-	-	T	T	T	G	A	-	A	A	A	G	A	-	-	G	A
16S11	T	A	C	A	A	C	G	C	A	A	A	A	T	G	C	A	T	-	-	-	-	-	-	A	-	-	-	-	-	T	T	G	C	A	-	G	T	A	A	G	-	-	G	A
16S12	T	A	C	C	G	T	G	C	A	A	T	A	T	G	C	A	T	-	-	-	-	-	-	A	A	T	-	-	T	T	-	G	T	A	-	A	T	A	A	G	-	-	G	A
16S13	T	A	C	C	A	T	G	C	G	A	A	A	T	A	C	A	T	-	-	-	-	-	-	A	-	-	-	-	-	T	T	G	T	T	-	G	T	A	A	G	-	-	A	A
16S14	T	A	C	C	A	T	G	C	A	A	T	A	T	G	C	A	T	-	-	-	-	-	-	A	A	-	-	-	T	T	T	G	T	A	-	G	T	A	A	G	-	-	G	A
