# Supplementary material for: Genetic analysis of Cryptozona siamensis (Stylommatophora, Ariophantidae) populations in Thailand using the mitochondrial 16S rRNA and COI sequences
Source: PLoS One. 2020 Sep 14;15(9):e0239264. doi: 10.1371/journal.pone.0239264 (PMC7489551; doi:10.1371/journal.pone.0239264)
Supplement: S4 Table — (PDF) [file pone.0239264.s004.pdf]

S4 Table. Haplotype frequency based on 16S rRNA sequences in each population.

Population Code	Haplotype													
	16S1	16S2	16S3	16S4	16S5	16S6	16S7	16S8	16S9	16S10	16S11	16S12	16S13	16S14
Phitsanulok						1						3		
Phetchabun					4									
Pathum Thani					4									
Nakhon Pathom												3	1	
Chon Buri										3				
Loei				2	1							1		
Nong Bua Lam Phu				4										
Chaiyaphum				2	2									
Maha Sarakham				1	2			2						
Buri Ram				3				1						
Chiang Rai			2	1										
Chiang Mai							1							1
Nan			3											
Uttaradit					1									
Chumphon				2							3			
Surat Thani							3							
Pattani			1											
Langkawi	4	1							2					
