# Supplementary material for: Genetic analysis of Cryptozona siamensis (Stylommatophora, Ariophantidae) populations in Thailand using the mitochondrial 16S rRNA and COI sequences
Source: PLoS One. 2020 Sep 14;15(9):e0239264. doi: 10.1371/journal.pone.0239264 (PMC7489551; doi:10.1371/journal.pone.0239264)
Supplement: S5 Table — (PDF) [file pone.0239264.s005.pdf]

S5 Table. Fifty-seven variable sites across the 14 haplotypes of *C. siamensis* based on COI sequences.

Haplotype	Nucleotide positions																			
	13	30	37	42	49	72	105	126	132	135	141	156	162	168	216	219	222	225	228	252
CO1	A	C	A	T	C	G	T	C	T	A	T	A	A	C	A	T	T	A	A	G
CO2	A	C	A	T	C	G	T	C	T	A	T	A	A	T	A	T	T	A	G	G
CO3	A	T	A	G	C	A	C	T	T	A	C	G	G	T	G	A	T	A	G	A
CO4	A	T	A	T	C	G	T	C	T	G	T	A	A	T	A	T	C	A	A	G
CO5	A	T	A	T	T	G	T	T	T	A	T	A	A	T	A	T	T	A	A	G
CO6	A	T	A	G	C	A	C	T	C	A	C	G	G	T	T	A	T	A	G	A
CO7	A	T	A	T	C	G	T	C	T	A	T	A	A	T	A	C	T	A	G	G
CO8	A	T	A	T	C	G	T	C	T	G	T	A	A	T	A	T	C	A	A	G
CO9	A	T	A	T	T	G	T	C	T	A	T	A	A	T	A	T	T	G	A	G
CO10	A	T	A	T	T	G	T	C	T	A	T	A	A	T	A	T	T	G	A	G
CO11	A	T	A	T	T	G	T	C	T	A	T	A	A	T	A	T	T	G	A	G
CO12	A	T	A	T	C	G	T	C	T	G	T	A	A	T	A	T	C	A	A	G
CO13	A	T	A	T	C	G	T	C	T	A	T	A	A	T	A	T	T	A	G	G
CO14	G	T	G	T	T	G	T	C	T	A	T	A	A	T	A	T	T	G	A	G

S5 Table. (continued)

Haplotype	Nucleotide positions																			
	**259**	**262**	**264**	**277**	**297**	**300**	**312**	**315**	**321**	**333**	**336**	**339**	**348**	**351**	**360**	**372**	**375**	**396**	**399**	**402**
CO1	T	C	G	G	T	A	G	A	A	G	C	G	C	T	G	A	T	C	A	C
CO2	T	C	G	G	T	A	G	A	T	A	C	G	C	T	G	A	T	T	A	C
CO3	C	T	T	A	T	G	T	T	C	A	T	G	T	T	A	A	C	T	G	T
CO4	T	C	C	G	T	A	G	A	T	G	C	G	T	T	G	A	T	T	A	C
CO5	T	C	C	G	T	A	G	A	T	G	T	G	C	T	G	A	T	C	A	C
CO6	C	T	T	A	T	T	T	T	C	A	C	A	T	C	A	G	C	T	G	T
CO7	T	C	C	G	T	G	G	G	T	G	C	G	C	T	G	A	T	C	A	C
CO8	T	C	C	G	T	G	G	G	T	G	C	G	T	T	G	A	T	T	A	C
CO9	T	C	C	G	T	G	G	G	T	A	C	G	C	T	G	A	T	C	A	T
CO10	T	C	C	G	T	G	G	G	T	A	C	G	C	T	G	A	T	C	A	T
CO11	T	C	C	G	C	G	G	G	T	A	C	G	C	T	G	A	T	C	A	T
CO12	T	C	C	G	T	G	G	G	T	G	C	G	T	T	G	A	T	T	A	C
CO13	T	C	C	G	T	G	G	G	T	G	C	G	C	T	G	A	T	C	A	C
CO14	T	C	C	G	T	G	G	G	T	A	C	G	C	T	G	A	T	C	A	T

S5 Table. (continued)

Haplotype	Nucleotide positions																		
	**405**	**411**	**417**	**426**	**429**	**462**	**474**	**480**	**489**	**492**	**507**	**517**	**543**	**549**	**552**	**558**	**577**			
CO1	G	A	A	A	T	A	T	G	G	A	G	G	G	T	A	G	T			
CO2	A	A	A	A	C	A	T	C	G	A	G	G	G	C	A	G	T			
CO3	A	G	C	G	T	G	A	A	A	G	G	G	A	T	G	T	C			
CO4	A	A	A	A	T	A	T	C	G	G	G	C	G	C	A	T	T			
CO5	A	A	A	A	T	A	T	A	G	G	G	G	G	C	A	G	T			
CO6	A	G	C	G	C	G	T	A	A	G	G	G	A	T	G	T	C			
CO7	A	A	A	A	T	A	T	C	G	G	G	G	G	T	A	G	T			
CO8	A	A	A	A	T	A	T	C	G	G	G	G	G	C	A	T	T			
CO9	A	A	A	A	T	A	T	G	G	G	A	G	G	C	A	G	T			
CO10	A	A	A	A	T	A	T	G	G	G	A	G	G	C	A	T	T			
CO11	A	A	A	A	T	A	T	A	G	G	A	G	G	C	A	G	T			
CO12	A	A	A	A	T	A	T	G	G	G	G	G	G	C	A	T	T			
CO13	A	A	A	A	T	A	T	C	G	A	G	G	G	C	A	G	T			
CO14	A	A	A	A	T	A	T	G	G	G	A	G	G	C	A	G	T			
