# Supplementary material for: Genetic analysis of Cryptozona siamensis (Stylommatophora, Ariophantidae) populations in Thailand using the mitochondrial 16S rRNA and COI sequences
Source: PLoS One. 2020 Sep 14;15(9):e0239264. doi: 10.1371/journal.pone.0239264 (PMC7489551; doi:10.1371/journal.pone.0239264)
Supplement: S6 Table — (PDF) [file pone.0239264.s006.pdf]

S6 Table. Haplotype frequency based on COI sequences in each population.

**Population Code**	**Haplotype**													
	**CO1**	**CO2**	**CO3**	**CO4**	**CO5**	**CO6**	**CO7**	**CO8**	**CO9**	**CO10**	**CO11**	**CO12**	**CO13**	**CO14**
Phitsanulok			3						1					
Phetchabun		3			1									
Pathum Thani		4												
Nakhon Pathom										1		3		
Chon Buri							2						1	
Loei	2	1	1											
Nong Bua Lam Phu	4													
Chaiyaphum	2	2												
Maha Sarakham	1	2				2								
Buri Ram			3			1								
Chiang Rai	1	1		1										
Chiang Mai					1						1			
Nan				3										
Uttaradit		1												
Chumphon	2							2						1
Surat Thani					3									
Pattani				1										
